# Identification of the Oncogenic Role of MSH2 in the Stemness and Progression of Glioma Through Regulating Wnt Signaling Pathway

**DOI:** 10.1002/cam4.70993

**Published:** 2025-06-30

**Authors:** Jun Liu, Jiayu Chen, Lianglei Jiang

**Affiliations:** ^1^ Department of Neurosurgery, Taihe Hospital Hubei University of Medicine Shiyan Hubei China; ^2^ Department of Neurosurgery, Hubei Provincial Clinical Research Center for Central Nervous System Repair and Functional Reconstruction, Taihe Hospital Hubei University of Medicine Shiyan Hubei China; ^3^ Department of Information Resources, Taihe Hospital Hubei University of Medicine Shiyan Hubei China; ^4^ Department of Neurosurgery Wuhan Union Hospital Wuhan Hubei China

**Keywords:** drug sensitivity, glioma, MSH2, tumor development

## Abstract

**Background:**

Glioma is one of the most aggressive brain tumors, and its progression is often associated with stemness maintenance and therapy resistance. The role of MSH2 in glioma remains largely unclear.

**Methods:**

We analyzed public datasets and clinical samples to assess MSH2 expression and its clinical relevance. Functional assays in vitro and in vivo were performed to investigate the effects of MSH2 knockdown on glioma cell behavior. Mechanistic studies were conducted to explore downstream signaling pathways and stemness regulation.

**Results:**

MSH2 was found to be significantly upregulated in glioma tissues and cell lines, and its high expression correlated with poor prognosis. Silencing MSH2 inhibited cell proliferation, migration, and tumor growth, while promoting apoptosis and G2 cell cycle arrest. Mechanistically, phospho‐kinase screening and rescue experiments suggested that MSH2 promotes glioma progression via activation of the Wnt/β‐catenin signaling pathway. Furthermore, MSH2 knockdown suppressed the expression of stemness markers, impaired sphere formation, and sensitized glioma cells to cisplatin treatment.

**Conclusions:**

Our study identifies MSH2 as an oncogenic factor in glioma, which drives stemness and progression through regulation of the Wnt/β‐catenin pathway, and may serve as a potential therapeutic target.

**Abbreviations**
EMT: epithelial‐mesenchymal transition
FBS: fetal bovine serum
GBM: glioblastoma
MMR: mismatch repair
MSH2: Mut S homolog 2
MSI: microsatellite instability
OD: optical density
PBS: phosphate‐buffered saline
PBST: PBS Tween
PCR: polymerase chain reaction
WB: Western Blot

## 1 Introduction

Gliomas, mostly arising from glial cells, are neuroepithelial tumors and are the most common intracranial primary central nervous system malignant tumor. According to epidemiological statistics, the incidence of glioma in adults is about 8/100,000, accounting for 46%–70% of central nervous system tumors, of which glioblastoma (GBM), the most malignant type of glioma, accounts for about 14.5% of all glioma cases [1, 2, 3]. Due to the characteristics of GBM such as high malignancy, high growth invasiveness, and easy metastasis and recurrence, the prognosis of GBM patients is very poor, and the median survival time is about 2 years [4]. At present, surgery is still the most important and commonly used means of glioma treatment. The highly invasive nature of GBM, however, poses great difficulties in surgical treatment [5, 6]. In recent years, with the development of genomics technology, a large number of molecular biological studies are rapidly updating the understanding of glioma, and the 2021 WHO Classification of Tumors of the Central Nervous System has also brought more molecular characteristics into account [7, 8]. Thanks to these, molecular targeted therapy has also gradually become one of the conventional treatments for glioma; however, its improvement in the prognosis of GBM patients is still very limited [9, 10, 11]. Therefore, in‐depth exploration of the molecular mechanism of glioma development will help to further promote the treatment of glioma to the direction of precision medicine, which is of great significance to improve the life treatment and prognosis of patients.

Alterations in the DNA mismatch repair (MMR) pathway play an important role in tumor development [12]. Loss or deficiency of MMR pathways is present in many types of malignant tumors, which is represented by the altered expression of MMR proteins [13, 14, 15, 16]. The MMR system consists of a variety of genes and proteins. To date, at least six MMR genes have been isolated from human cells, three of which are MutS homologs: Mut S homolog 2 (MSH2), MSH6, and MSH3, and the other three are MutL homologs, namely MLH1, PMS1, and PMS2. The primary manifestation of MMR system dysfunction is the accumulation of DNA errors and genomic instability, that is, microsatellite instability (MSI), which plays an important role in the mechanism of tumor formation in a variety of malignant tumors such as endometrial cancer, colorectal cancer, and gastric cancer [17, 18, 19]. In addition, the proteins encoded by MMR genes also play an important role in the regulation of cytotoxicity induced by DNA‐damaging drugs [20, 21, 22]. MSH2 is one of the earliest discovered and most studied MMR genes, which is able to perform the MMR process by forming Mut S‐α heterodimers, thereby ensuring microsatellite stability. Mutations in MSH2 can be detected in a variety of malignancies such as gastric cancer, endometrial cancer, and colorectal cancer [23, 24, 25]. However, recent studies have shown that MSH2 expression and function in tumors may be tissue‐dependent; for example, a pan‐cancer study showed that MSH2 expression was significantly upregulated in a variety of tumors and was significantly correlated with immune infiltration and patients' prognosis [26]. Therefore, this work focused on revealing the expression and functional role of MSH2 in glioma progression.

The Wnt signaling pathway plays a pivotalness and driving tumor progression in glioma [27, 28]. Aberrant activation of Wnt/β‐catenin signaling sustains glioma stem cells (GSCs), a subpopulation characterized by self‐renewal capacity, tumor‐initiating potential, and notably, therapeutic resistance—a hallmark of treatment failure and recurrence in GBM [29, 30]. These GSCs are considered key contributors to glioma recurrence and aggressive behavior, particularly in GBM. However, the upstream regulatory mechanisms linking MSH2 to Wnt‐driven stemness remain poorly understood. This study addresses a critical gap by investigating whether MSH2 modulates Wnt activity to foster glioma stemness and chemoresistance, thereby offering insights into dual‐targeted strategies to disrupt the GSC niche and overcome therapeutic resistance, ultimately improving clinical outcomes.

In this study, we aimed to investigate the oncogenic role of MSH2 in glioma. By integrating public datasets, clinical samples, and in vitro and in vivo experiments, we comprehensively evaluated the expression pattern and clinical significance of MSH2 in glioma. Functional studies were conducted to determine the effects of MSH2 knockdown on cell proliferation, apoptosis, migration, and tumorigenicity. Furthermore, mechanistic investigations identified the Wnt/β‐catenin signaling pathway as a downstream effector of MSH2. We also explored the influence of MSH2 on glioma stemness and cisplatin sensitivity. Our findings reveal a novel role of MSH2 in promoting glioma progression and maintaining stem‐like properties via Wnt signaling activation, providing new insights into its potential as a therapeutic target.

## 2 Materials and Methods

### 2.1 Bioinformatics Analysis

Gene expression and survival analyses were performed using datasets from the GEO and CGGA databases. For differential expression analysis, the GEO dataset GSE153908 (https://www.ncbi.nlm.nih.gov/geo/query/acc.cgi?acc=GSE153908), which includes glioma and normal brain tissue samples, was utilized. The raw microarray data were normalized using the “affy” package in *R*, and differentially expressed genes were identified using the “limma” package. For survival analysis, RNA‐seq expression profiles and corresponding clinical data were obtained from the CGGA database (http://www.cgga.org.cn/). The FPKM expression matrices from two CGGA RNA‐seq cohorts (mRNAseq_693 and mRNAseq_325) were merged, and batch effects were removed using the “combat” function from the “sva” package in R. The resulting data were used to analyze the association between MSH2 expression and patient survival, including overall survival and disease‐free survival. In addition, correlation analyses between MSH2 and glioma stemness‐related markers (CD44, EPCAM, and SOX2) were also performed using the CGGA database.

### 2.2 Cell Culture

Three glioma cell lines including HEB, U87, and U251 cells were purchased from the National Infrastructure of Cell Line Resource (Beijing, China). The SHG‐44 cell line was purchased from Pricella (Wuhan, China). HEB cells were cultured with RPMI 1640 (w/o Hepes) 15% NBS. U87 and U251 cells were cultured with MEM‐EBSS. While SHG‐44 cells were cultured with H‐DMEM, 10% fetal bovine serum (FBS) and 1% P/S. All cells were cultured in a humidified incubator containing 5% CO_2_ at 37°C, and the culture medium was changed every 3 days.

### 2.3 Immunohistochemistry (IHC) Staining

This study was approved by the Ethical Committee of Taihe Hospital, Hubei University of Medicine. All the tissue donors signed the informed consent form; the baseline clinical information was provided as well.

Tissue microarray analysis involving 88 glioma cancer tissues and 32 adjacent normal tissues were used for IHC staining. Paraffin‐embedded samples were placed in the oven at 65°C for 30 min, subsequently dewaxed in xylene. Next, samples were rehydrated in gradient alcohol and repaired in the citrate repair solution (pH = 6.0) under 180°C for 5 min. Samples were washed 3 times with 1× phosphate‐buffered saline (PBS) and PBS Tween (PBST) buffer was added. The endogenous peroxidase was blocked with 3% H_2_O_2_ and 5% serum separately. The samples were incubated with primary anti‐MSH2 antibody (1:200, Abcam, USA) overnight at 4°C. In addition, the samples were incubated with secondary antibody (goat anti‐rabbit IgG H&L (HRP), 1:400, Abcam, USA) overnight at 4°C. The samples were washed with 1× PBST 3 times, and DAB solution was added to stain in the dark for 5 min. After washing, samples were counterstained with hematoxylin (Baso, China) for 15 s. Finally, the samples were sealed with neutral gum, and stain results were observed using a microscope. The stain degree and the positive cells were documented.

### 2.4 Construction of Lentiviral Vector and Lentivirus Infection

Targeting MSH2 RNA interference (RNAi) lentiviral vector was prepared. The synthesized DNA oligonucleotides were annealed to form double‐stranded DNA, and the target gene RNAi was inserted into the BR‐V‐108 vector (Shanghai Yibeirui Biomedical Science and Technology Co. Ltd., China). Then, the recombinant plasmid carrying target sequences was transfected into *Escherichia coli* . DNA sequencing analysis was used to confirm the vector expression by polymerase chain reaction (PCR). Positive cloned plasmids were extracted according to the EndoFree Maxi Plasmid Kit (Sigma‐Aldrich Co. LLC., China) instructions. Lentivirus were packaged with prepared plasmids for infection. The U87 and U251 cells were digested with trypsin, inoculated in a 10 cm cell culture dish when the cell density was 5 × 10^6^/15 mL, and cultured in a 37°C with 5% CO_2_ incubator. When the cell density reached 80%, the transfection reagent was added. After culturing in a 37°C with 5% CO_2_ incubator for 72 h, the cell supernatant was collected. The supernatant was centrifuged at 10000 rpm. Infection efficiency was detected by fluorescence microscopic.

### 2.5 Quantitative Real Time PCR (qPCR)

Trizol reagent (Sigma, USA) was used for total RNA extraction. The quality of total RNA was evaluated by Nanodrop 2000C spectrophotometer (Thermo Fisher Scientific, USA). High‐quality cDNA was obtained by reverse transcription using Promega M‐MLV kit according to the factory's protocol. qPCR was carried out using SYBR Green mastermix Kit (Vazyme, China) and applying ABI VII7 Real‐Time PCR System (ABI, USA). The *F* = 2^−△△Ct^ method was used to evaluate the relative expression of MSH2 RNA, with GAPDH as an internal reference. Details of the primer sequence were shown as follows.

MSH2 forward primer sequence (5′‐3′): ACTGTCTGCGGTAATCAAGTT.

MSH2 reverse primer sequence (5′‐3′): CTGACTGCTGCAATATCCAA.

GAPDH forward primer sequence (5′‐3′): TGACTTCAACAGCGACACCCA.

GAPDH reverse primer sequence (5′‐3′): CACCCTGTTGCTGTAGCCAAA.

### 2.6 Western Blot (WB) Assay

Total protein was extracted from the lentivirus‐infected U87 and U251 cells by utilizing the IP cell lysate and PMSF (100:1) on ice at 4°C. BCA Protein Assay Kit (HyClone‐Pierce, USA) was utilized to detect the total protein concentration. Protein was separated using SDS‐PAGE with SDS‐Acryl/Bis (Tanon, China). The transfer electrophoresis device (Tanon, China) was used to transfer protein onto the PVDF membrane. Subsequently, the TBST solution containing 5% skim milk was added to block the PVDF membrane at room temperature for 1 h. The primary antibodies, including anti‐MSH2 (1:1000, Abcam, USA), anti‐Wnt (1:1000, Abcam, USA), anti‐β‐Catenin (1:2000, Proteintech, China), anti‐E‐Cadherin (1:3000, Proteintech, China), anti‐N‐Cadherin (1:1000, Proteintech, China), anti‐SOX2 (1:1000, Proteintech, China), anti‐CD44 (1:2000, BOSTER, China) and anti‐GAPDH (1:30000, Proteintech, China) were added to the membranes separately. Next, the unsealed PVDF membranes were incubated at room temperature for 2 h. After this, the membranes were washed with TBST solution three times. The PVDF membranes were incubated at room temperature for 1 h when the secondary antibody (Goat Anti‐Mouse/Goat Anti‐Rabbit, 1:3000, Beyotime, Germany) was added. The membranes were washed with TBST solution 3 times again. Finally, the blots were visualized by Millipore's Immobilon Western Chemistry HRP Substrate kit (Merck, USA) according to factory's instructions.

### 2.7 Celigo Cell Counting Assay

After digesting by trypsin, the lentivirus‐infected U87 and U251 cells were seeded on a 96‐well plate with 2 × 10^3^ cell/well in triplicate separately. Cell counting was detected by using a Celigo image cytometer (Nexcelom Bioscience, USA) for five consecutive 5 days. The number of cells with fluorescence was captured. The data was statistically plotted, and the 5‐day cell proliferation curve was drawn.

### 2.8 Cell Apoptosis and Cell Cycle

Flow cytometry was utilized to detect cell apoptosis and the cell cycle.

For cell apoptosis, lentivirus‐infected U87 and U251 cells were seeded into a 6‐well plate in triplicate separately, and the cells were cultured for 5 days. The cells were treated with 11 μg/mL cisplatin (MACKLIN, China). The cell supernatant was collected in a 5 mL centrifuge tube. The cells were collected in the same 5 mL centrifuge tube after washing with D‐Hank's once. The cells were digested by trypsin. As well as the cells were collected in the same 5 mL centrifuge tube. After centrifuging, the cells were washed and precipitated with D‐Hank's solution once. The cells were centrifuged at 1500 rpm for 3 min. Additionally, the 1 × binding buffer was added to wash the cells in order to form precipitation. Next, precipitation was centrifuged at 1500 rpm for 3 min. 1 mL 1 × cell staining buffer was resuspended cell precipitation. Subsequently, 5 μL annexin V‐APC (eBioscience, USA) and 5 μL propidium iodide (Sigma, USA) were separately added to stain for 15 min, keep away from light. The cell apoptosis was detected by the flow cytometer (Millipore, USA).

For cell cycle, lentivirus‐infected U87 and U251 cells were seeded into 6 cm dishes in triplicate separately. When the cells grew to a coverage rate of 80%, the supernatant of the cell culture was discarded. After washing and digesting, the cells were collected in a 5 mL centrifuge tube. Subsequently, the cells were centrifuged for 5 min, and the supernatant was discarded. Cells were washed with precooled PBS (pH = 7.2) at 4°C. Then, the cells were centrifuged at 1500 rpm for 5 min. Cells were fixed with 70% precooled ethanol at 4°C for 1 h. After centrifuging, 1.5 mL of cell staining solution (40 × PI mother liquor: 100× RNase mother liquor: 1× PBS = 25:1:1000) was added to suspend. Cell cycle was measured by the flow cytometer (Millipore, USA).

### 2.9 CCK8 Assay

After digesting, lentivirus‐infected U87 and U251 cells were seeded into a 96‐well plate with 4 × 10^3^ cell/well in triplicate separately. The cells were cultured at 37°C with 5% CO_2_ in an incubator. Next, 10 μL CCK‐8 reagent (Sigma, USA) was added into the plates. Subsequently, the optical density (OD) was measured by using the microplate reader (Tecan infinite, Switzerland) at 450 nm. Finally, the dose response curve was drawn.

### 2.10 Wound‐Healing Assay

After digesting, the complete culture medium was added to resuspend the cell suspension, and the lentivirus‐infected U87 and U251 cells were seeded into a 96‐well plate with 5 × 10^4^ cell/well in triplicate separately as well. In addition, the plates were cultured in a 37°C with 5% CO_2_ incubator. The next day, the low concentration serum culture medium was changed. Scratches were formed by using a scratch tool aligned with the median of the 96‐well plate and gently pushed from bottom to top. The serum‐free medium was used to wash the cells 3 times. After this, 0.5% FBS serum medium was added to each plate that was cultured at 37°C with 5% CO_2_ in the incubator. The cells were captured by using Cellomics (Thermo, USA) at 0 h and 24 h. The cell migration rate of each group was calculated as well.

### 2.11 Transwell Assay

The upper transwell chamber was filled with 100 μL serum‐free medium, and the chamber was placed in an incubator for 2 h. The 600 μL culture medium containing 30% FBS was added to the lower chamber. Lentivirus‐infected U87 and U251 cells were seeded into a 24‐well plate with 5 × 10^4^ cell/well. The cells were cultured at 37°C with 5% CO_2_ in the incubator for 4 h. The chamber was inverted on the absorbent paper, and the nontransfer cells were slightly removed with cotton. The 400 μL Giemsa dye solution was added to the plate for staining. Finally, photographs were captured through using a microscope (Olympus, Japan).

### 2.12 Human Phospho‐Kinase Array

Human Phospho‐Kinase Array Kit was performed according to the factory's instructions. Briefly, the 1.0 mL array buffer was added into the array per well and the array was rocked for 1 h at room temperature. The array was incubated overnight at 5°C on a rocking platform shaker. Later, the array was washed with 1× wash buffer 3 times. Diluted detection antibody cocktail A 1.0 mL was added into each well for Part A membranes. Diluted detection antibody cocktail B 1.0 mL was added into each well for Part B membranes. Subsequently, the membranes were incubated on a rocking platform for 2 h at room temperature. The membranes were washed with 1× wash buffer 3 times. Diluted Streptavidin‐HRP 1.0 mL was added into each well. Then, the membranes were transferred to the appropriate wells. In addition, the membranes were incubated on a rocking platform for 30 min at room temperature. After washing with 1× wash buffer 3 times, the membranes were arranged on a sheet protector and the Chemi Reagent Mix was added to the membranes. Finally, the membranes were exposed to X‐ray film for 10 min.

### 2.13 Xenograft Tumor Model In Vivo

A total of 10 female 4‐week‐old BALB/c nude mice were purchased from GEMPHARMATECH Co. Ltd. (China) and randomly divided into shCtrl or shMSH2 group. About 4 × 10^6^ cells were subcutaneously injected into the mice. The tumor growth was observed after 5 days. Tumor sizes were documented once a week. On the 20th day, the mice were anesthetized by intraperitoneal injection of 0.7% pentobarbital sodium (10 μL/g, Sigma, USA). Average fluorescence intensity was captured by utilizing the Caliper IVIS Lumina II (BERTHOLD TECHOLOGIES, Germany). After removing, the tumors were weighed and measured. Tumor volume was calculated by this equation *V* = *π*/6 × *L* × *W* ^2^. In addition, tumor tissues were immunohistochemically stained with primary antibody anti‐Ki67 (1:50, Abcam, USA) and secondary antibody (Goat anti‐rabbit IgG H&L (HRP), 1:400, Abcam, USA). This animal experiment was approved by the Ethical Committee of Taihe Hospital, Hubei University of Medicine (No. 2023KS16).

### 2.14 3D Sphere Formation Assay

Glioma cells were digested into single‐cell suspensions and seeded in ultralow attachment 6‐well plates (Corning, USA) at a density of 1 × 10^4^ cells per well in serum‐free DMEM/F12 medium supplemented with B27 supplement (1×, Gibco, USA), 20 ng/mL EGF, and 20 ng/mL bFGF. The cells were cultured at 37°C in a humidified incubator with 5% CO_2_ for 7–10 days. Tumor spheres were then imaged under a microscope, and sphere size was quantified for analysis.

### 2.15 Statistical Analysis

SPSS software and GraphPad Prism software were used to perform the data analysis. Data were exhibited as the mean and standard deviation for continuous variables. GraphPad Prism 6.01 software (GraphPad Software Inc.) was used to draw relevant charts. Student's *t*‐test and *χ* ^2^ test were used to analyze the statistical difference. Statistical analysis between multiple groups were performed by using SPSS 22.0 (IBM, USA) with one‐way ANOVA. Kaplan–Meier method was performed to draw the survival curve. A *p* value < 0.05 that should be considered to indicate a statistically significant difference.

## 3 Results

### 3.1 MSH2 Is Upregulated in Glioma and Correlated With Poor Prognosis of Patients

**FIGURE 1:**
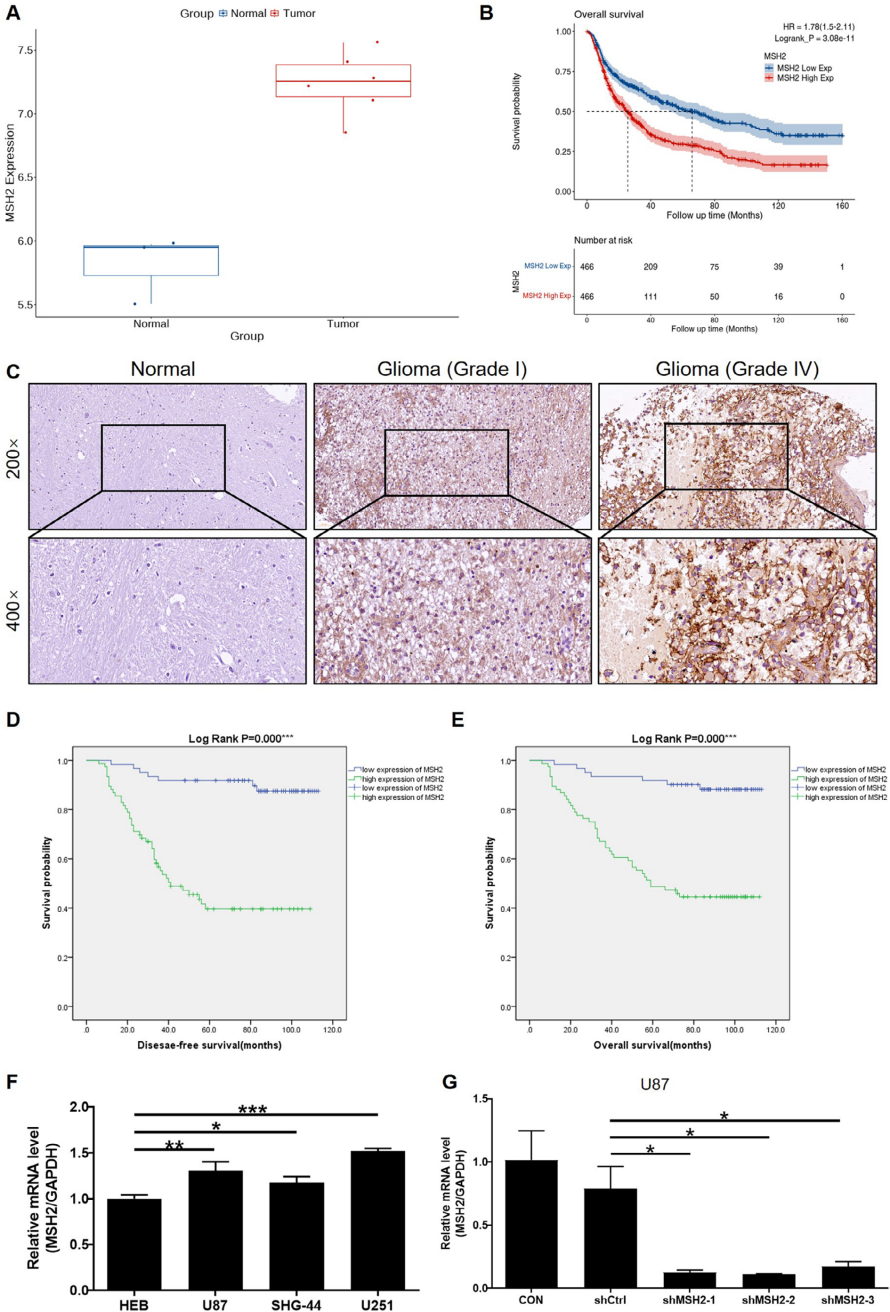
MSH2 expression is upregulated in glioma and correlates with poor prognosis. (A) Differential expression of MSH2 in glioma tissues and normal brain tissues was analyzed based on GSE153908 dataset. (B) The relationship between MSH2 expression and patients' prognosis is examined through Kaplan–Meier survival analysis. (C) IHC staining was performed to analyze the protein level of MSH2 in glioma tissues with different tumor grade and normal brain tissues. (D, E) Kaplan–Meier survival analysis was used to establish the linkage between MSH2 expression and disease‐free survival and overall survival of glioma patients. (F) The endogenous mRNA level of MSH2 in human brain glial cells HEB and glioma cell lines including U87, SHG‐44 and U251. (G) The knockdown efficiencies of MSH2 by the various shRNAs were examined by the detection of MSH2 expression by qPCR. Data were shown as mean with standard deviation (SD) (*n* ≥ 3). *p* < 0.05 was considered to be statistically significant. *p < 0.05, **p < 0.01, ***p < 0.001.

As the initial step to reveal the role of MSH2 in glioma progression, the expression pattern and potential in predicting patient's survival were analyzed based on the GSE153908 dataset and CGGA database, respectively. The results showed the upregulation of MSH2 in glioma tissues compared with normal ones (Figure 1A), as well as the correlation between MSH2 high expression and poorer survival probability (Figure 1B). Subsequently, a tissue microarray containing 88 tumor tissues and 32 normal brain tissues was used for molecular expression evaluation. As shown by the representative images in Figure 1C and the statistical data in Table 1, the expression of MSH2 was significantly upregulated in glioma tissues in comparison with normal brain tissues. It was also demonstrated that the increase of the malignancy degree of tumor (represented by the tumor grade) is accompanied by the elevation of MSH2 expression (Figure 1C, Tables 2 and 3). Moreover, we also found a positive correlation between higher risk of tumor reoccurrence and high MSH2 expression (Tables 2 and 3). Furthermore, Kaplan–Meier survival analysis indicated the significant association between high MSH2 expression and disease‐free survival (Figure 1D) as well as overall survival (Figure 1E) of glioma patients. For example, as for overall survival, MSH2‐low patients (*n* = 61) showing average survival periods of 87.9 months versus 60.4 months in MSH2‐high expression patients (*n* = 76) highlights its prognostic significance. Except for the higher expression in glioma tissues, relatively higher endogenous expression of MSH2 was also observed in glioma cell lines including U87, U251, and SHG‐44 compared with normal human astrocytes HEB cells (Figure 1F). All above results indicated the potential role of MSH2 in the promotion of glioma development, which is worthy of further investigation.

**TABLE 1:** Expression patterns of MSH2 in glioma tissues and normal tissues revealed in immunohistochemistry analysis.

MSH2 expression	Tumor tissue		Normal tissue	
	Cases	Percentage	Cases	Percentage
Low	61	44.5%	27	84.3%
High	76	55.5%	5	15.7%

*Note:* *p* < 0.001.

**TABLE 2:** Relationship between MSH2 expression and tumor characteristics in patients with glioma.

Features	No. of patients	MSH2 expression		*p*
		Low	High	
All patients	137	61	76	
Gender				
Male	88	36	52	0.255
Female	49	25	24	
Grade				
I	17	13	4	< 0.001
II	57	33	24	
III	46	14	32	
IV	17	1	16	
Tumor recurrence				
No	61	48	13	< 0.001
Yes	76	13	63	

**TABLE 3:** Relationship between MSH2 expression and tumor characteristics in patients with glioma analyzed by Spearman rank correlation analysis.

Tumor characteristics	Index	
Grade	Spearman correlation	0.235
	Significance (two tailed)	0.006
	*n*	136
Tumor recurrence	Spearman correlation	0.619
	Significance (two tailed)	< 0.001
	*n*	137

### 3.2 Silencing of MSH2 Inhibits Cell Proliferation and Induces Cell Apoptosis

**FIGURE 2:**
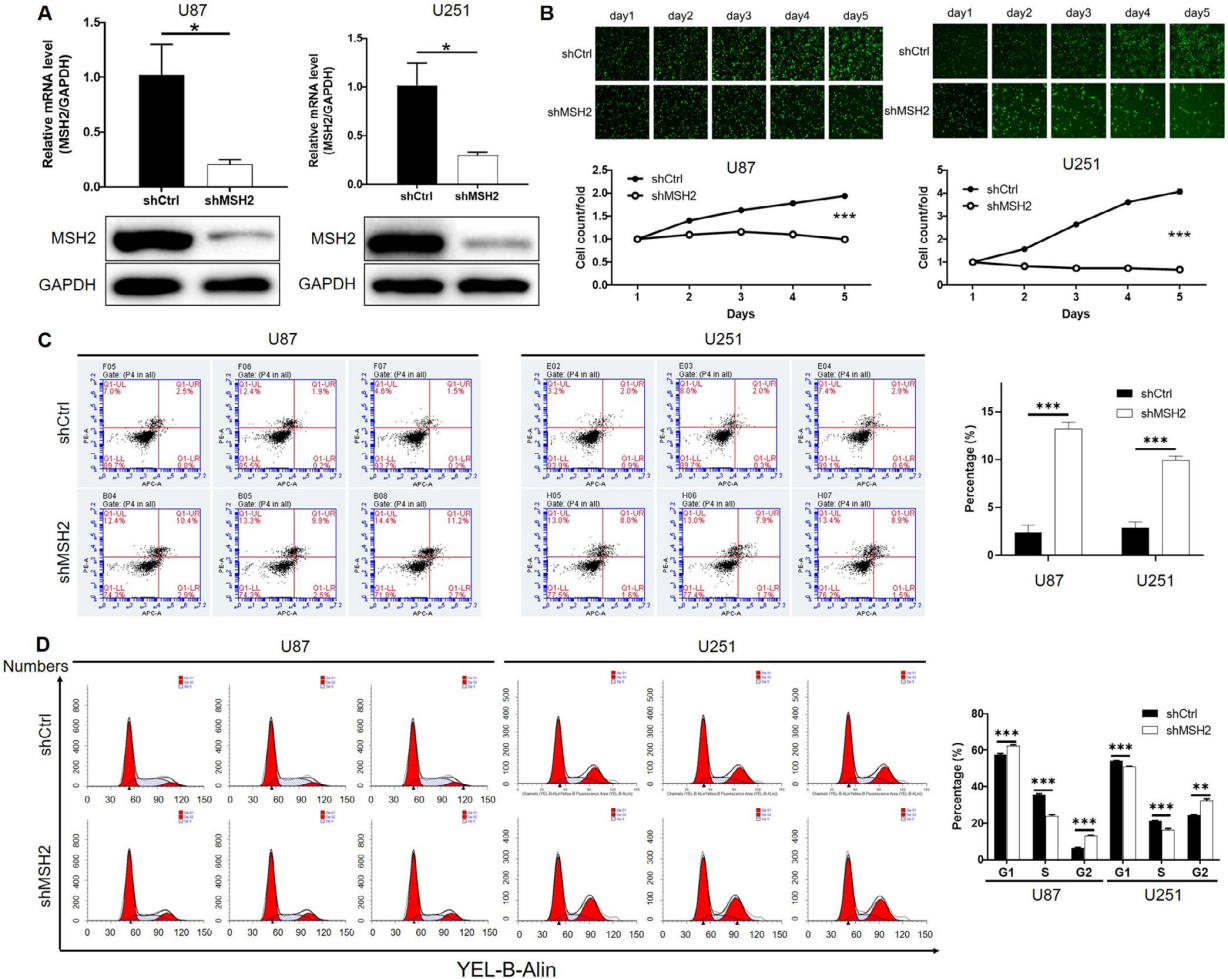
MSH2 knockdown inhibits glioma cell growth in vitro. (A) The knockdown of MSH2 by shMSH2 in U87 and U251 cells was confirmed by qPCR and western blot. (B) The inhibition of cell proliferation by MSH2 knockdown was confirmed by celigo cell counting assay. (C) The enhancement of cell apoptosis induced by MSH2 knockdown was visualized by flow cytometry. (D) The change of cell cycle distribution in U87 and U251 cells with or without MSH2 knockdown was examined by flow cytometry. Data were shown as mean with standard deviation (SD) (*n* ≥ 3). *p* < 0.05 was considered to be statistically significant. *p < 0.05, **p < 0.01, ***p < 0.001.

For carrying out in vitro studies to visualize the regulatory functions of MSH2 in glioma development, MSH2 knockdown glioma cell lines were constructed through transfection with lentivirus expressing shMSH2. Since the results of qPCR showed that all 3 shRNAs targeting MSH2 silencing exhibited excellent efficacy in U87 cells, the best of them (shMSH2‐2) was selected for application in all following experiments without further explanation (Figure 1G). Following the successful knockdown of MSH2 in U87 and U251 cells, which is revealed by the apparent decline of the mRNA and protein level of MSH2 in shMSH2 groups (Figure 2A), a series of in vitro experiments were carried out to estimate the changes in cell proliferation, cell apoptosis, and cell cycle distribution upon MSH2 knockdown. As shown by the results of the celigo cell counting assay in Figure 2B, the growth rate of both U87 and U251 cells was significantly slowed down after the knockdown of MSH2. Conversely, the cell apoptosis rate was significantly elevated in shMSH2 groups, which was also in consistent with the inhibited cell proliferation (Figure 2C). A similar trend was also observed in SHG‐44 cells (Figure S1). Otherwise, the cell cycle of glioma cells with or without MSH2 knockdown was also detected and compared. Although there were slight differences in the results of the G1 phase, it was demonstrated that MSH2 knockdown induced the decrease of S phase cells and the increase of G2 phase cells (Figure 2D). Collectively, MSH2 silence may inhibit cell proliferation through inducing cell apoptosis and G2 arrest of the cell cycle.

### 3.3 MSH2 Knockdown Inhibits Migratory Ability of Glioma Cells

**FIGURE 3:**
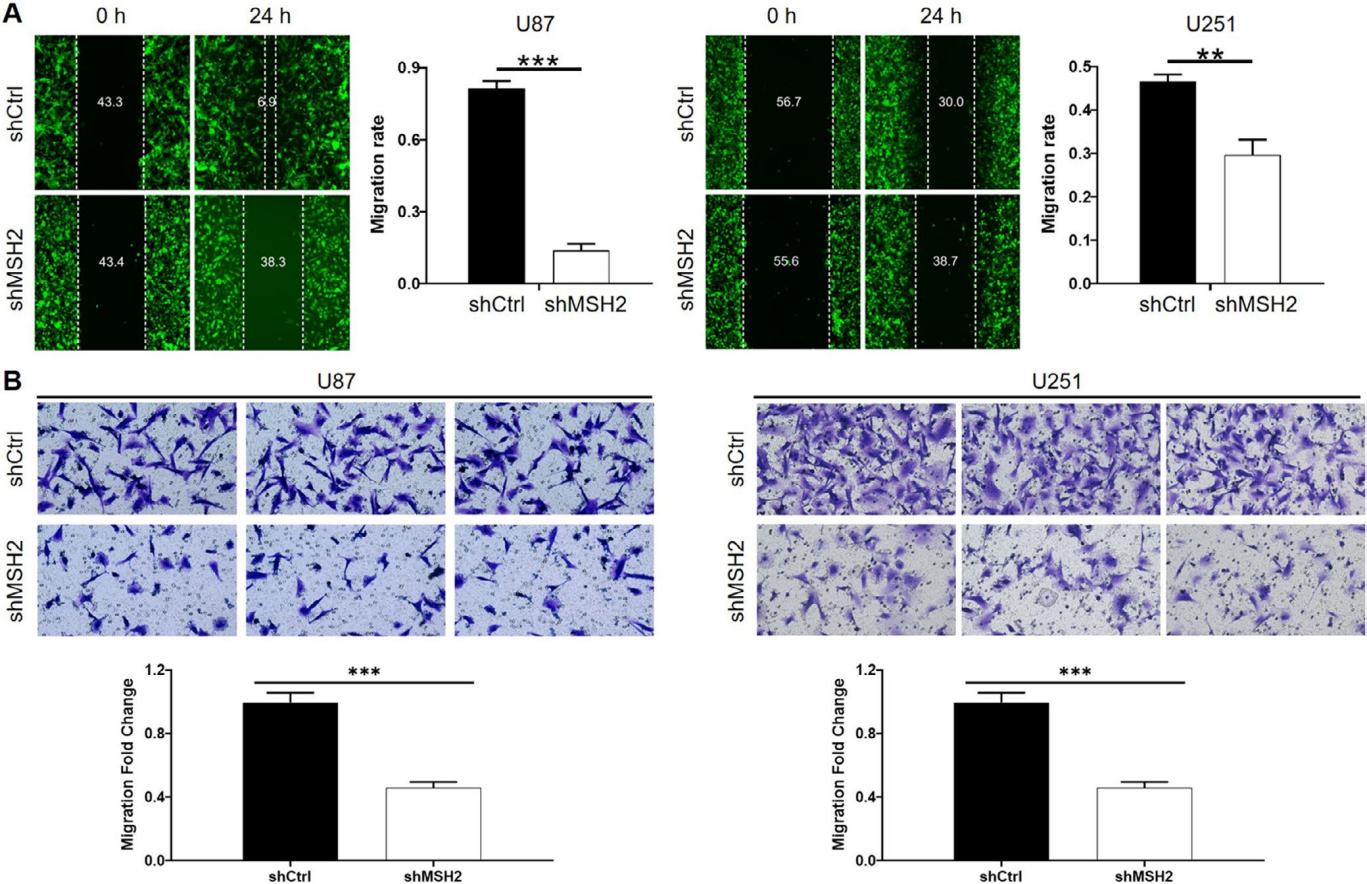
MSH2 knockdown inhibits glioma cell migration. Cell motility was tested by wound‐healing assay (A) and transwell assay (B) for U87 and U251 cells with or without MSH2 knockdown. Data were shown as mean with standard deviation (SD) (*n* ≥ 3). *p* < 0.05 was considered to be statistically significant. **p < 0.01, ***p < 0.001.

Besides the rapid proliferation, the migration ability of cancer cells, which may result in tumor metastasis, is also an important hallmark of cancer. Therefore, shCtrl and shMSH2 cells were subjected to scratch assay and transwell assay for the evaluation of cell migration capacity. As shown in Figure 3A,B, slower wound healing and fewer migrated cells in the shMSH2 groups illustrated the inhibition of cell migration ability upon MSH2 knockdown. Therefore, the outcomes of both assays indicated that knockdown of MSH2 may be an efficient strategy to decrease the migratory activity of glioma cells.

### 3.4 MSH2 Knockdown Inhibited Glioma Cell Growth In Vivo

**FIGURE 4:**
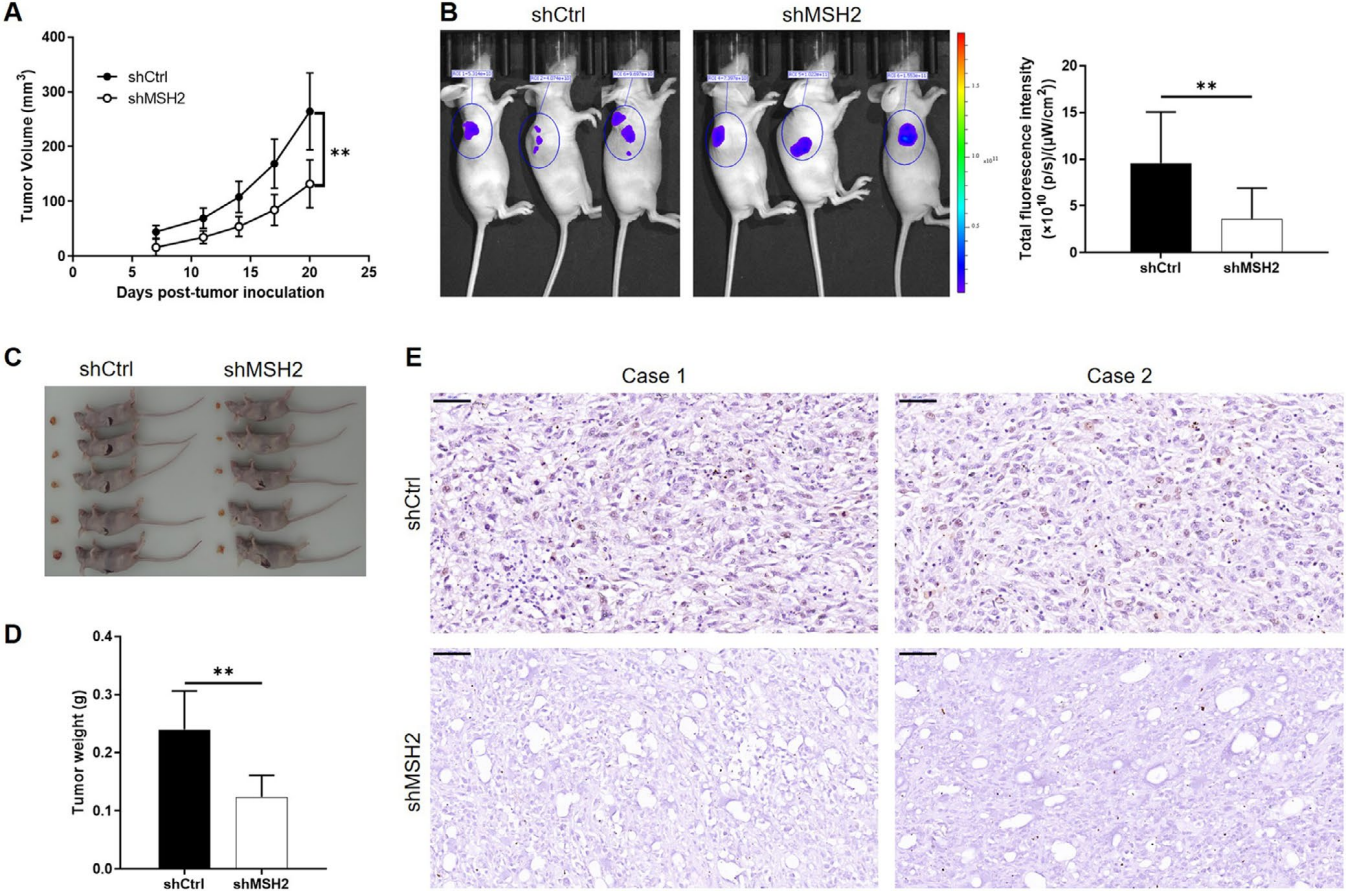
MSH2 knockdown suppress tumor growth in vivo. Mice xenograft models were constructed through the subcutaneous injection of SHG‐44 cells transfected with shCtrl or shMSH2. Then the tumor size was measured at indicated time points and tumor volume was calculated accordingly (A). (B) Before sacrificing the mice, they were anesthetized and subjected to living animal fluorescence imaging for evaluating the tumor burden. After animal sacrifice, xenografts were harvested for taking photos (C) and weighing (D). (E) Ki67 expression in xenografts collected from both groups of mice was detected by IHC staining. Data were shown as mean with standard deviation (SD) (*n* ≥ 3). *p* < 0.05 was considered to be statistically significant. **p < 0.01.

For launching animal studies, BALB/c nude mice were subcutaneously injected with SHG‐44 cells infected with shCtrl or shMSH2 for forming xenografts. During the 20 days of animal experiments, we have been measuring and calculating the tumor volume and found that the growth of transplanted tumor in the shMSH2 group was significantly slower (Figure 4A). In situ fluorescence imaging of the xenografts, using the fluorescence intensity resulting from the GFP labeled on the lentivirus vector as a representative of tumor burden, also suggested that smaller final tumors were formed by shMSH2 cells in corresponding groups of animals (Figure 4B). After terminating the animal experiments and sacrificing the mice, xenografts were removed, collected for taking photos (Figure 4C) and weighing (Figure 4D), which again indicated the inhibition of tumor growth by MSH2 knockdown. Finally, on a molecular level, the suppression of glioma growth by MSH2 knockdown was also revealed by the downregulation of Ki67, a widely used index for tumor growth activity (Figure 4E). In summary, all the above results identified MSH2 as an important participant in the development and progression of glioma, with an unclear mechanism.

### 3.5 MSH2 May Regulate the Development of Glioma Through the Mediation by Wnt Signaling Pathway

**FIGURE 5:**
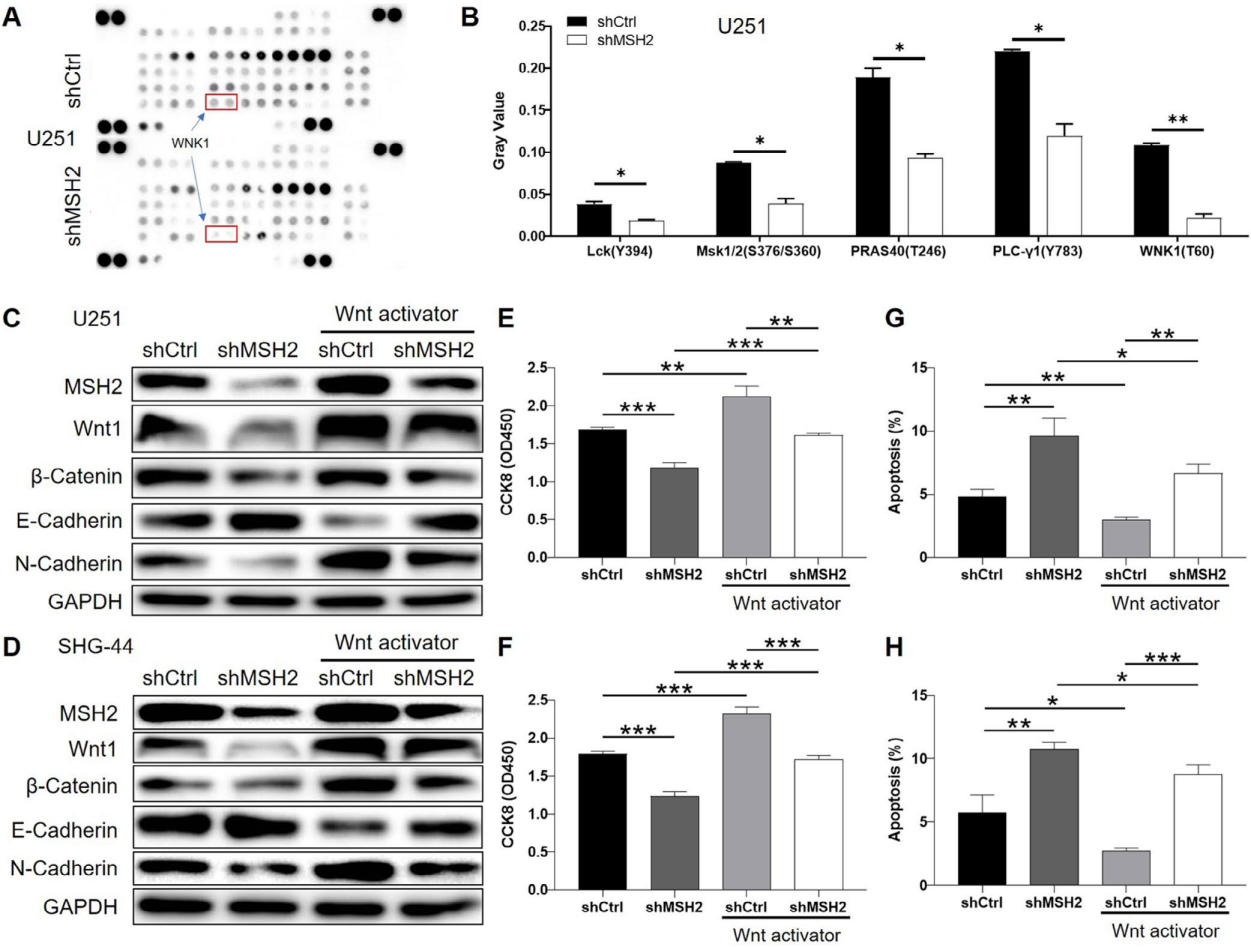
MSH2 regulates the development of glioma through activating Wnt/β‐catenin signaling pathway. (A) A Human Phospho‐Kinase Array‐Membrane was applied for screening potentially downstream signaling pathway involved in MSH2‐related regulation of glioma. (B) The gray value of several differentially expressed phospho‐kinases identified by the array was shown. (C, D) The rescue experiments between MSH2 knockdown and Wnt activator treatment were performed on U251 (C) and SHG‐44 (D) cells in the respect of Wnt pathway related proteins and EMT biomarkers. (E, F) Rescue experiments between MSH2 knockdown and Wnt activator treatment were performed on U251 (E) and SHG‐44 (F) cells in the respect of cell proliferation. (G, H) Rescue experiments between MSH2 knockdown and Wnt activator treatment were performed on U251 (G) and SHG‐44 (H) cells in the respect of cell apoptosis. Data were shown as mean with standard deviation (SD) (*n* ≥ 3). *p* < 0.05 was considered to be statistically significant. *p < 0.05, **p < 0.01, ***p < 0.001.

For further insight into the mechanism by which MSH2 influences the phenotypes of glioma cells, a Human Phospho‐Kinase Array‐Membrane was applied for screening potentially involved downstream signaling pathways (Figure 5A and Table S1). Among the differentially expressed phospho‐kinases, we noticed the downregulation of the phosphorylation level of WNK1, which is a positive regulator of the Wnt signaling pathway [31] (Figure 5B). Therefore, we further examined the regulation of Wnt1 and β‐catenin levels by MSH2 in U251 and SHG‐44 cells, both of which indicated that MSH2 knockdown may inhibit the activation of the Wnt/β‐catenin signaling pathway, which could be partially recovered by using a Wnt activator HY‐135516 (Figure 5C,D). Moreover, it was also proved that Wnt/β‐catenin‐mediated regulation of epithelial‐mesenchymal transition (EMT) by MSH2 [32] (Figure 5C,D). Furthermore, the treatment of shMSH2 cells with HY‐135516 could attenuate the shMSH2‐induced inhibition of cell proliferation and the enhancement of cell apoptosis (Figure 5E–H). Collectively, the mechanistic study identified the Wnt signaling pathway as a potential downstream of MSH2 in the regulation of glioma.

### 3.6 MSH2 Knockdown Impairs Glioma Stemness and Enhances Cisplatin Sensitivity

**FIGURE 6:**
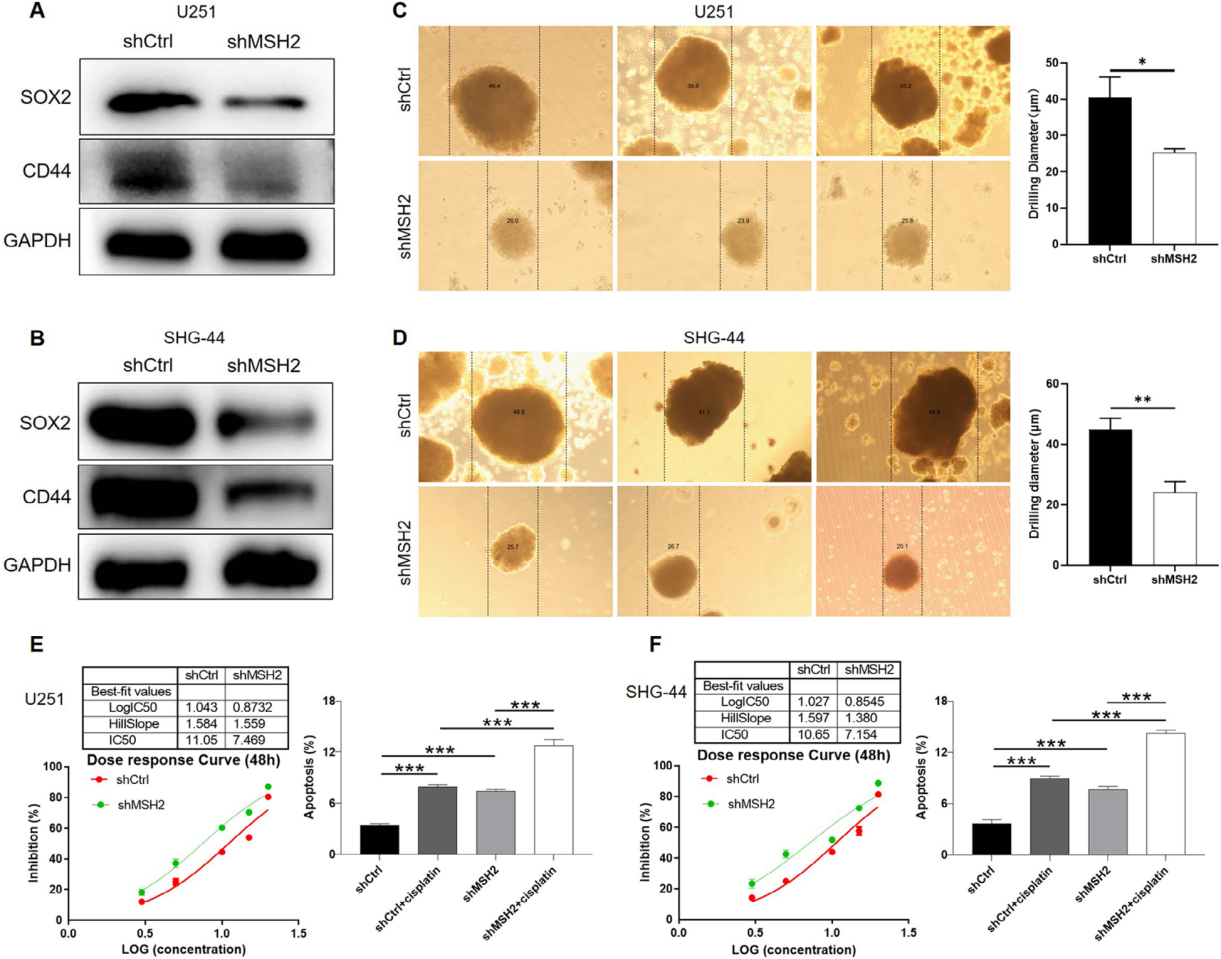
MSH2 knockdown increase the sensitivity of glioma cells to cisplatin. (A, B) The expression of stemness‐related proteins including CD44 and SOX2 was detected in shCtrl and shMSH2 groups of U251 (A) and SHG‐44 (B) cells. (C, D) The sphere‐forming abilities of U251 (C) and SHG‐44 (D) cells were assessed by a 3D sphere formation assay. (E, F) The effects of MSH2 on cisplatin IC50 and cisplatin‐induced cell apoptosis were evaluated in U251 (E) and SHG‐44 (F) cells. Data were shown as mean with standard deviation (SD) (*n* ≥ 3). *p* < 0.05 was considered to be statistically significant. *p < 0.05, **p < 0.01, ***p < 0.001.

To explore the potential role of MSH2 in maintaining glioma stemness, we first analyzed the correlation between MSH2 and classical stemness markers using the CGGA database. The results showed that MSH2 expression was positively correlated with CD44, EPCAM, and SOX2 (Figure S2). Consistently, it was revealed that knockdown of MSH2 markedly decreased the protein levels of CD44 and SOX2 in U251 (Figure 6A), SHG‐44 (Figure 6B), and U87 cells (Figure S3A). Furthermore, 3D sphere formation assays demonstrated that MSH2 depletion significantly reduced the size of tumor spheres in all three cell lines, indicating a loss of stem‐like properties (Figure 6C,D, Figure S3B). In addition, we assessed the response of glioma cells to cisplatin upon MSH2 knockdown. As shown in Figure 6E,F, silencing MSH2 decreased the IC50 of cisplatin and enhanced cisplatin‐induced apoptosis in both U251 and SHG‐44 cells. Taken together, these data suggest that MSH2 contributes to the maintenance of glioma stemness and mediates resistance to cisplatin.

## 4 Discussion

DNA is prone to errors during replication, while DNA MMR proteins such as MLH1, PMS2, MSH2, and MSH6 are important repair genes during DNA replication to ensure the fidelity of the replication process. Mutations in MMR genes can lead to an autosomal dominant disorder called Lynch syndrome, in which patients are at higher risk of colorectal and endometrial cancer [33, 34, 35]. The reason for this is that deletion of at least one of the MMR protein coding genes including MLH1, MSH2, MSH6, and PSM2 can lead to loss of MMR function and difficulty in correcting mismatches during DNA replication, and ultimately DNA MSI. MSI in key regions can affect normal signaling pathway transduction, gene translation and transcription and protein modification, and ultimately cause the generation of tumor cells and lead to tumorigenesis [35]. MSH2 was first discovered and one of the main focuses of researchers when studying MMR genes. Numerous studies have shown that pathogenic variants affecting MSH2 are one of the causes of Lynch syndrome and cause a variety of different carcinogenic risks [36]. Liccardo et al. showed that miR‐137, which can target the 3′ UTR of MSH2, is able to reversely regulate its expression in colorectal cancer, and dysregulation of the process may play a role in promoting the development of Lynch syndrome‐related tumors [37]. On the other hand, the regulatory role of MSH2 itself in the development of tumors has also attracted the attention of scientists in recent years, however its function is still obscure. For example, McCoy et al. and Sharma et al. found that loss or reduced expression of MSH2 was significantly associated with poor prognosis in prostate cancer patients, which was not clearly associated with the MSI [38, 39]. Zhang et al. showed that loss of expression of both circLIFR and MSH2 inhibited cisplatin sensitivity in bladder cancer cells, respectively. The complex formed by circLIFR and MSH2, on the other hand, is able to promote the stability of p73 by hindering the interaction between MutSα and ATM, which in turn induces apoptosis and increases the cisplatin sensitivity of bladder cancer cells [40]. A recent pan‐cancer bioinformatics analysis showed that MSH2 expression was upregulated in most types of tumors and was significantly correlated with patient prognosis, indicating that MSH2 may play an important role in tumor occurrence, development and immune infiltration [26]. However, till now, there are still very few studies concerning the regulatory role of MSH2 in tumor progression. Herein, our study found that MSH2 protein level was significantly upregulated in glioma tissues and associated with higher degree of malignancy and poorer prognosis, which is contrary to its expression pattern in prostate cancer but in consistent with the most recent pan‐cancer study [26]. Subsequent in vitro and in vivo loss‐of‐function experiments confirmed the tumor‐suppressing profile of MSH2 downregulation in glioma, which blocks cell proliferation, disturbs cell migration while inducing cell apoptosis and cell cycle arrest.

Beyond its role in promoting general malignant phenotypes, our study revealed that MSH2 may facilitate glioma progression by activating the Wnt/β‐catenin signaling pathway. Phospho‐kinase profiling identified decreased phosphorylation of WNK1, a known positive regulator of Wnt signaling, in MSH2‐deficient cells. Correspondingly, knockdown of MSH2 reduced Wnt1 and β‐catenin expression, which could be partially restored by a Wnt activator. Reactivation of Wnt signaling also attenuated the MSH2 knockdown‐induced inhibition of proliferation, promotion of apoptosis, and suppression of EMT‐related features. These findings suggest that MSH2 promotes glioma progression at least in part through the activation of Wnt/β‐catenin signaling.

Wnt signaling is widely recognized for its pivotal role in glioma biology. Previous studies have shown that various oncogenes, such as SRPK1 and TMEM64, promote glioma proliferation, migration, and invasion by activating Wnt/β‐catenin signaling [41, 42]. Furthermore, Wnt signaling is deeply intertwined with glioma stemness [43]. For instance, DAB2IP deficiency was found to upregulate Wnt‐mediated NLGN3 secretion, which in turn transformed neighboring glioma cells into cancer stem cells (CSCs) and contributed to tumor aggressiveness [44]. Similarly, treatment with cyanidin was shown to suppress glioma stem cell (GSC) viability via downregulating β‐catenin and Wnt target genes such as MYC and TWIST1 [45]. These studies reinforce the notion that the Wnt/β‐catenin pathway plays a central role in regulating CSC properties in glioma.

Crucially, CSCs have also been recognized as a major driving force behind glioma chemoresistance. It has been demonstrated that glioma cells with high stemness exhibit stronger resistance to temozolomide (TMZ) and other chemotherapeutic agents [46]. For instance, RIP2 was shown to induce chemoresistance by enhancing CSC properties via activation of the NF‐κB pathway, and blocking this axis sensitized glioma cells to TMZ [47]. Moreover, under hypoxic conditions, Galectin‐8 was found to promote CSC maintenance via autophagy activation, further contributing to treatment resistance [48]. Notably, several studies have now suggested a convergence of Wnt signaling, CSC maintenance, and resistance. For example, the lncRNA LINC00839, overexpressed in glioma stem cells, was found to promote radiation resistance by activating Wnt signaling through c‐Src‐mediated phosphorylation of β‐catenin, and this resistance could be overcome by cotreatment with Wnt inhibitors [29].

In line with these findings, our data demonstrate that MSH2 depletion led to downregulation of glioma stemness markers CD44 and SOX2 and reduced sphere‐forming ability in multiple cell lines. Furthermore, MSH2 knockdown enhanced cisplatin‐induced cytotoxicity, reducing IC50 values and increasing apoptotic rates. Importantly, the suppressive effects of MSH2 knockdown on glioma growth and survival could be attenuated by pharmacological activation of Wnt signaling, highlighting the functional relevance of this pathway downstream of MSH2. Taken together, our results suggest that MSH2 promotes glioma stemness and chemoresistance by sustaining Wnt/β‐catenin activity, thereby positioning MSH2 as a potential therapeutic target in glioma treatment strategies aimed at eradicating CSCs and overcoming drug resistance.

Several limitations of this study should be acknowledged. First, although consistent phenotypic changes were observed across multiple glioma cell lines, intercell line variability, particularly between U87 and U251 cells, may reflect underlying molecular heterogeneity that warrants further investigation. Second, the in vivo experiments were conducted using a subcutaneous xenograft model in female nude mice, which does not fully recapitulate the intracranial tumor microenvironment or account for sex‐based biological differences. Third, while WNK1 was identified as a potential upstream link between MSH2 and Wnt signaling, additional kinases were also affected, and further quantitative proteomic studies are needed to comprehensively map the downstream network. Lastly, although high MSH2 expression correlates with poor patient prognosis, we cannot exclude the possibility that MSH2 acts as a surrogate marker for glioma grade rather than an independent prognostic driver, which will require future clinical and multivariate analyses for clarification.

In summary, our study demonstrates that MSH2 is upregulated in glioma and associated with poor prognosis. Functional experiments revealed that MSH2 promotes glioma cell proliferation, migration, and tumor growth both in vitro and in vivo. Mechanistically, MSH2 activates the Wnt/β‐catenin signaling pathway, contributing to EMT, stemness maintenance, and resistance to cisplatin. These findings identify MSH2 as a critical regulator of glioma progression and a potential therapeutic target for glioma treatment.

## Author Contributions

L.J. designed this program. J.L. operated the cell and animal experiments. J.L., J.C., and L.J. conducted the data collection and analysis. J.L. produced the manuscript, which was checked by J.C. and L.J. All the authors have confirmed the submission of this manuscript.

## Ethics Statement

This study was approved by the Ethical Committee of Taihe Hospital, Hubei University of Medicine. All the tissue donors signed the informed consent form; the baseline clinical information was provided as well.

## Consent

The authors have nothing to report.

## Conflicts of Interest

The authors declare no conflicts of interest.

## Supporting information


Figure S1.

Figure S2.

Figure S3.

Table S1.

## Data Availability

The data that support the findings of this study are available from the corresponding author upon reasonable request.
