# Supplementary figures and images for: Identification of the Oncogenic Role of MSH2 in the Stemness and Progression of Glioma Through Regulating Wnt Signaling Pathway
Source: Cancer Med. 2025 Jun 30;14(13):e70993. doi: 10.1002/cam4.70993 (PMC12209330; doi:10.1002/cam4.70993)

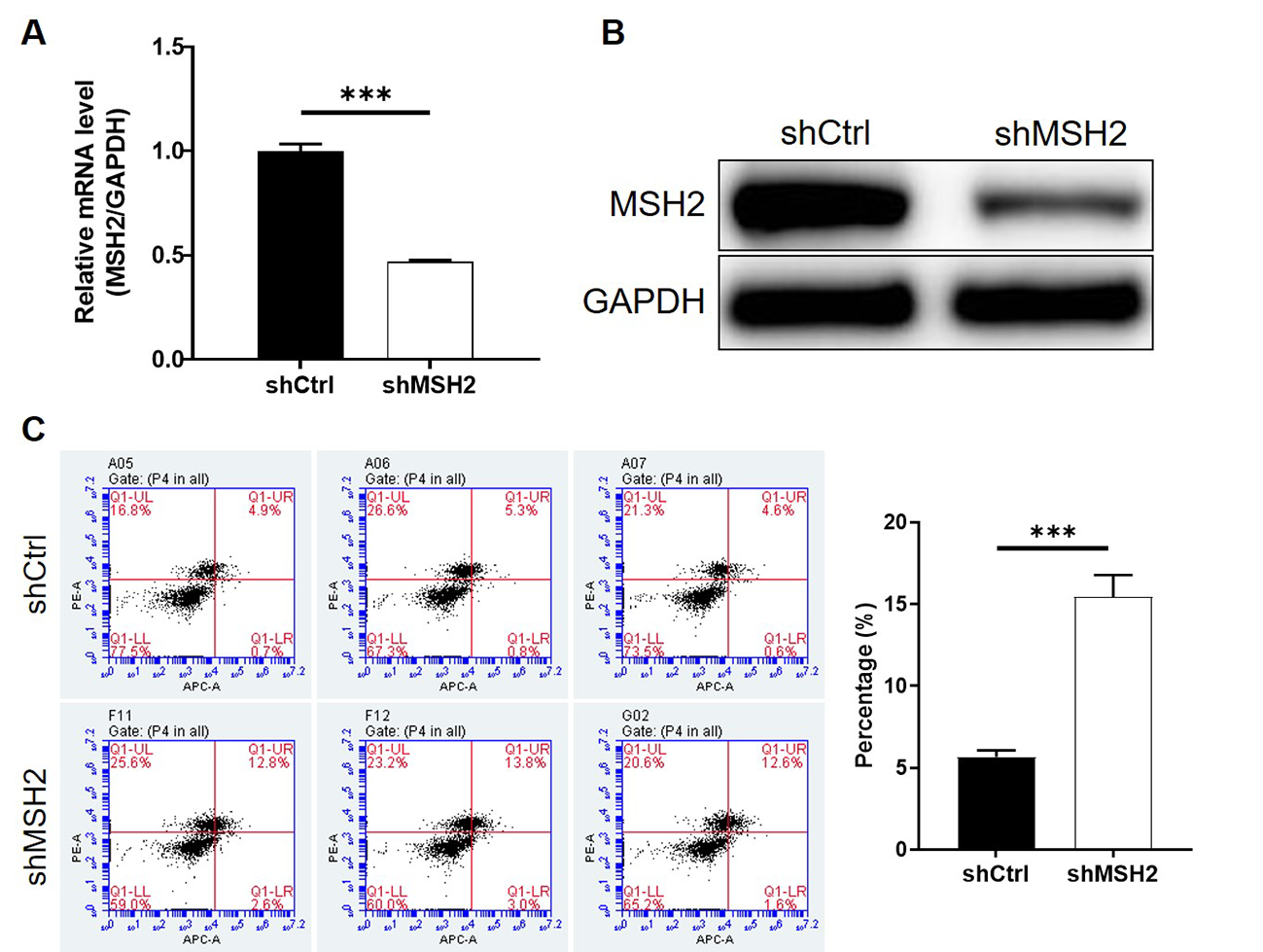

Supplement: Supplementary file 1 — Figure S1. [file CAM4-14-e70993-s001.tif]

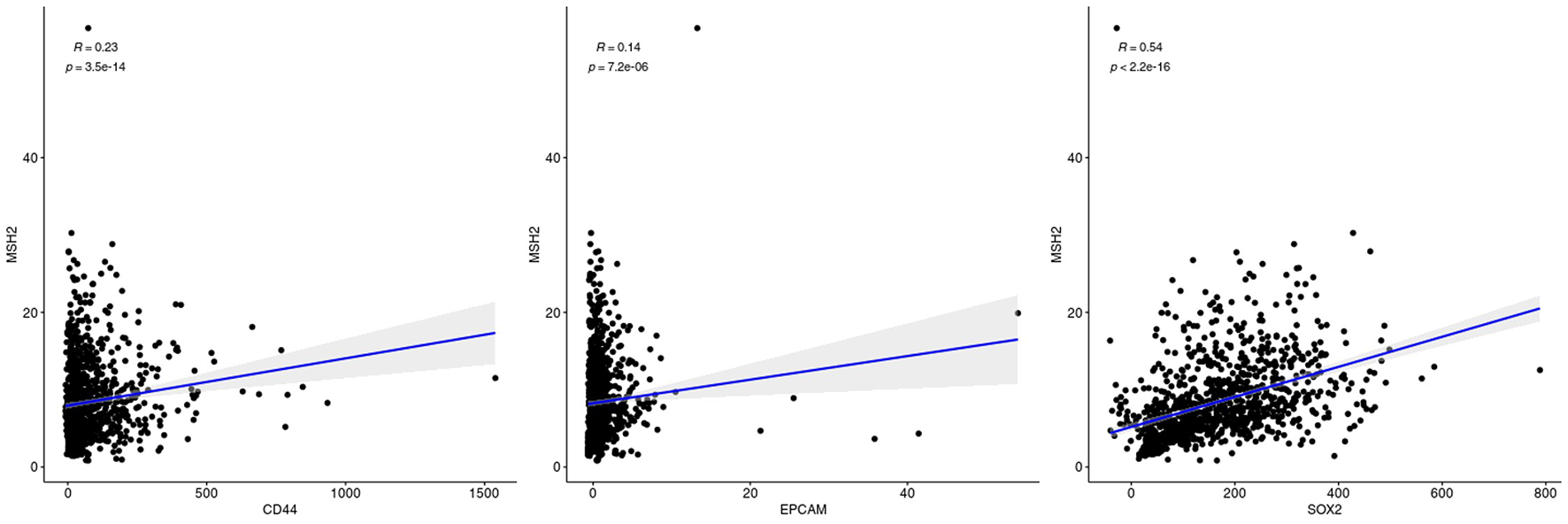

Supplement: Supplementary file 2 — Figure S2. [file CAM4-14-e70993-s004.tif]

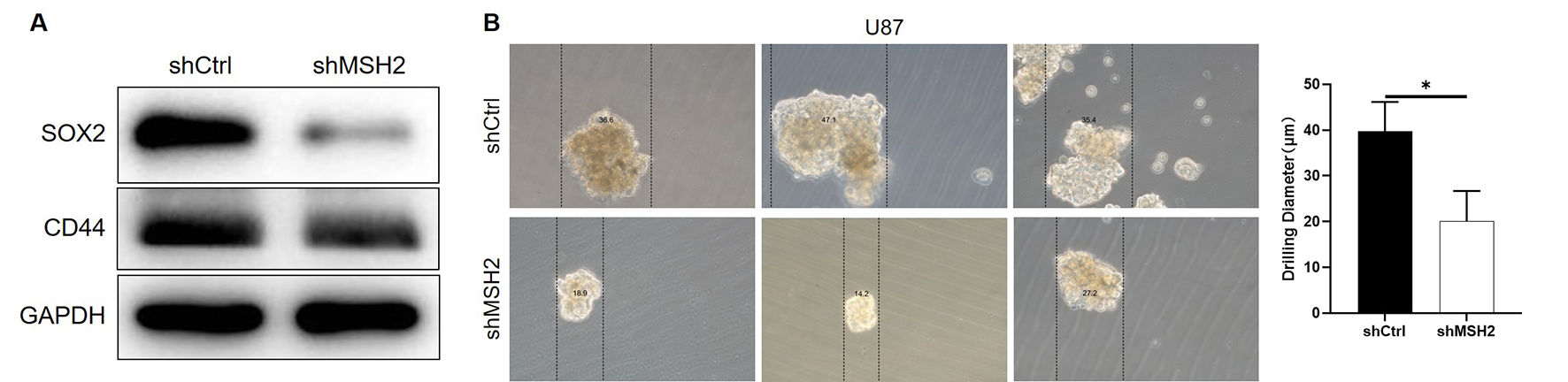

Supplement: Supplementary file 3 — Figure S3. [file CAM4-14-e70993-s003.tif]
