# Supplementary material for: Identification of the Oncogenic Role of MSH2 in the Stemness and Progression of Glioma Through Regulating Wnt Signaling Pathway
Source: Cancer Med. 2025 Jun 30;14(13):e70993. doi: 10.1002/cam4.70993 (PMC12209330; doi:10.1002/cam4.70993)
Supplement: Supplementary file 4 — Table S1. [file CAM4-14-e70993-s002.docx]

Table S1. The differentially expressed proteins identified by the Human Phospho-Kinase Array.

|  | **Target** | **AVERAGE Gray Value** | | **STDEV** | | **P VALUE** | **Up/Down** |
| --- | --- | --- | --- | --- | --- | --- | --- |
|  |  | **shCtrl/Positive Control** | **shMSH2/Positive Control** | **NC/Positive Control** | **KD/Positive Control** |  |  |
| **1** | **Akt1/2/3 (T308)** | 0.1000 | 0.0885 | 0.0060 | 0.0139 | 0.3956 | -11.51% |
| **2** | **Akt1/2/3 (S473)** | 0.0338 | 0.0445 | 0.0370 | 0.0009 | 0.7545 | 31.40% |
| **3** | **CREB (S133)** | 0.0932 | 0.1209 | 0.0035 | 0.0141 | 0.1139 | 29.73% |
| **4** | **EGFR (Y1086)** | 0.0278 | 0.0422 | 0.0014 | 0.0082 | 0.1337 | 51.65% |
| **5** | **eNOS (S1177)** | 0.1237 | 0.0806 | 0.0023 | 0.0161 | 0.0648 | -34.79% |
| **6** | **ERK1/2 (T202/Y204, T185/Y187)** | 0.1084 | 0.0587 | 0.0179 | 0.0074 | 0.0683 | -45.83% |
| **7** | **Chk-2 (T68)** | 0.1101 | 0.0922 | 0.0156 | 0.0180 | 0.4008 | -16.21% |
| **8** | **c-jun(S63)** | 0.0659 | 0.0449 | 0.0196 | 0.0170 | 0.3699 | -31.93% |
| **9** | **Fgr(Y412)** | 0.0716 | 0.0803 | 0.0007 | 0.0051 | 0.1386 | 12.11% |
| **10** | **GSK-3α/β (S21/S9)** | 0.3466 | 0.2558 | 0.0613 | 0.0012 | 0.2836 | -26.19% |
| **11** | **GSK-3β (S9)** | 0.2085 | 0.1150 | 0.0051 | 0.0146 | 0.0134 | **-44.84%** |
| **12** | **Hsp27 (S78/S82)** | 0.4424 | 0.2210 | 0.0275 | 0.1621 | 0.1973 | -50.05% |
| **13** | **p53(S15)** | 0.6720 | 0.6709 | 0.0289 | 0.0378 | 0.9772 | -0.16% |
| **14** | **p53(S46)** | 0.8546 | 0.7805 | 0.0022 | 0.0136 | 0.0168 | -8.67% |
| **15** | **p53(S392)** | 0.1497 | 0.0915 | 0.0399 | 0.0162 | 0.1958 | -38.87% |
| **16** | **JNK1/2/3 (T183/Y185, T221/Y223)** | 0.0898 | 0.0923 | 0.0090 | 0.0080 | 0.7994 | 2.75% |
| **17** | **Lck(Y394)** | 0.0384 | 0.0186 | 0.0040 | 0.0017 | 0.0236 | **-51.56%** |
| **18** | **Lyn(Y397)** | 0.0817 | 0.0558 | 0.0036 | 0.0100 | 0.0745 | -31.73% |
| **19** | **Msk1/2 (S376/S360)** | 0.0875 | 0.0389 | 0.0015 | 0.0084 | 0.0151 | **-55.52%** |
| **20** | **p70 S6K (T389)** | 0.0718 | 0.0706 | 0.0477 | 0.0238 | 0.9790 | -1.56% |
| **21** | **p70 S6K (T421/S424)** | 0.0418 | 0.0303 | 0.0065 | 0.0239 | 0.5784 | -27.52% |
| **22** | **PRAS40 (T246)** | 0.1891 | 0.0935 | 0.0156 | 0.0064 | 0.0153 | **-50.56%** |
| **23** | **p38α (T180/Y182)** | 0.1177 | 0.1368 | 0.0062 | 0.0027 | 0.0571 | 16.26% |
| **24** | **PDGF Rβ (Y751)** | 0.0676 | 0.0208 | 0.0163 | 0.0003 | 0.1539 | -69.20% |
| **25** | **PLC-γ1 (Y783)** | 0.2205 | 0.1191 | 0.0025 | 0.0208 | 0.0208 | **-45.98%** |
| **26** | **Src(Y419)** | 0.1365 | 0.0418 | 0.0109 | 0.0557 | 0.1421 | -69.36% |
| **27** | **PYK2 (Y402)** | 0.1799 | 0.1549 | 0.0076 | 0.0134 | 0.1494 | -13.87% |
| **28** | **RSK1/2 (S221/S227)** | 0.1952 | 0.1717 | 0.0377 | 0.0757 | 0.7321 | -12.04% |
| **29** | **RSK1/2/3 (S380/S386/S377)** | 0.1102 | 0.0365 | 0.0018 | 0.0382 | 0.1126 | -66.84% |
| **30** | **STAT2(Y689)** | 0.0773 | 0.0553 | 0.0189 | 0.0708 | 0.7119 | -28.51% |
| **31** | **STAT5a/b (Y694/Y699)** | 0.1411 | 0.1439 | 0.0273 | 0.0270 | 0.9273 | 1.98% |
| **32** | **WNK1(T60)** | 0.1084 | 0.0219 | 0.0034 | 0.0067 | 0.0037 | **-79.84%** |
| **33** | **Yes(Y426)** | 0.1694 | 0.2280 | 0.0114 | 0.1416 | 0.6190 | 34.56% |
| **34** | **STAT1 (Y701)** | 0.1613 | 0.1169 | 0.0192 | 0.0222 | 0.1655 | -27.52% |
| **35** | **STAT3 (Y705)** | 0.0210 | 0.0248 | 0.0044 | 0.0238 | 0.8432 | 18.29% |
| **36** | **STAT3 (S727)** | 0.1592 | 0.0876 | 0.0070 | 0.0832 | 0.3492 | -44.96% |
| **37** | **β-Catenin** | 0.2878 | 0.1858 | 0.0671 | 0.0975 | 0.3473 | -35.43% |
| **38** | **STAT6 (Y641)** | 0.0375 | 0.0282 | 0.0190 | 0.0208 | 0.6864 | -24.84% |
| **39** | **Hsp60** | 0.9053 | 0.8513 | 0.0077 | 0.0074 | 0.0191 | -5.96% |
